# Modeling brain network flexibility in networks of coupled oscillators: a feasibility study

**DOI:** 10.1038/s41598-024-55753-8

**Published:** 2024-03-08

**Authors:** Narges Chinichian, Michael Lindner, Serhiy Yanchuk, Tilo Schwalger, Eckehard Schöll, Rico Berner

**Affiliations:** 1 Institut für Theoretische Physik, Technische Universität Berlin, Berlin, Germany; 2 Psychiatry Department, Charité-Universitätsmedizin, Berlin, Germany; 3 Bernstein Center for Computational Neuroscience, Berlin, Germany; 4 Potsdam Institute for Climate Impact Research, Potsdam, Germany; 5 Institute of Mathematics, Humboldt Universität zu Berlin, Berlin, Germany; 6 School of Mathematical Sciences, University College Cork, Cork, Ireland; 7 Institute of Mathematics, Technische Universität Berlin, Berlin, Germany; 8 Department of Physics, Humboldt Universität zu Berlin, Berlin, Germany

**Keywords:** Computational neuroscience, Neuroscience, Applied physics, Statistical physics, thermodynamics and nonlinear dynamics, Computational neuroscience, Neuroscience, Applied physics, Statistical physics, thermodynamics and nonlinear dynamics

## Abstract

Modeling the functionality of the human brain is a major goal in neuroscience for which many powerful methodologies have been developed over the last decade. The impact of working memory and the associated brain regions on the brain dynamics is of particular interest due to their connection with many functions and malfunctions in the brain. In this context, the concept of brain flexibility has been developed for the characterization of brain functionality. We discuss emergence of brain flexibility that is commonly measured by the identification of changes in the cluster structure of co-active brain regions. We provide evidence that brain flexibility can be modeled by a system of coupled FitzHugh-Nagumo oscillators where the network structure is obtained from human brain Diffusion Tensor Imaging (DTI). Additionally, we propose a straightforward and computationally efficient alternative macroscopic measure, which is derived from the Pearson distance of functional brain matrices. This metric exhibits similarities to the established patterns of brain template flexibility that have been observed in prior investigations. Furthermore, we explore the significance of the brain’s network structure and the strength of connections between network nodes or brain regions associated with working memory in the observation of patterns in networks flexibility. This work enriches our understanding of the interplay between the structure and function of dynamic brain networks and proposes a modeling strategy to study brain flexibility.

## Introduction

The rapid growth of neuroscience in the past few decades has been the result of two major factors: the advancement in tools and techniques to acquire empirical and simulated brain data, and the introduction of new concepts to interpret this data. The network-based approach enables researchers to study brain structures represented by nodes on various scales (individual cells, neural ensembles, voxels, or areas) and their interactions (structural or functional)^1,2^. In neuroimaging-based network neuroscience [The term is used to refer to the application of graphs in the study of the brain^1^, brain regions identified by any given method of parcellation are typically regarded as the network’s nodes, while links can be defined either as white matter connections between brain regions (structural networks) or as statistical interdependencies between the time series of brain regions (functional networks)^3–9^.

A brain network in network neuroscience can be defined and studied along different dimensions and scales. A three-dimensional scale space with “spatial”, “temporal” and “topological” axes has been introduced by Betzel and Bassett^10^. The “spatial” scale of a network describes the granularity at which the network nodes and links are specified. The nodes, for example, can range from representing individual cells with synapses as links to representing brain regions with large-scale fiber tracts between the regions as links. The “temporal” scale, specifies the time-scale in which the brain dynamics is studied: a network can be based on quantities defined in smaller than millisecond time-resolution or quantities chosen to reflect centuries of evolution in the brains of species. Finally, the “topological” scale characterizes the scale in which different topological aspects of the network are captured and ranges between single node or links in a local context to the entire network in a global view.

While the study of single neurons or the whole brain as one entity are the two extremes of the spatial scale spectrum, many coarse-grained approaches of intermediate scale (often also called meso-scale approaches in network neuroscience) have been introduced and proved to be useful in the recent literature^11–16^.

Time dependent phenomena in the brain such as the progression of a disease^17–19^, transition between cognitive tasks^20,21^, learning^22–24^ and aging^19,25^ are best understood when dynamical network approaches are employed. Static measures of the network therefore need to be modified and generalized for a broader dynamic context^26^.

A widely used dynamical measure of the brain at topological meso-scales (groups of nodes in the network) is *flexibility*^27^, which is based on the detection of clusters of nodes that are co-active at given instances of time. The flexibility measure quantifies how often the network nodes are re-assigned to different clusters over time. In order to determine the clusters, various clustering/community-detection algorithms^20,21,28,29^ can be used. The flexibility measure has been found to be associated with mood, fatigue and novelty of experience^30^, aging^31^, learning^16^, executive function^20^ and mentalization^32^.

Despite the large extent of the literature on measuring the flexibility of the human brain, we lack a simplified mechanistic understanding of this measure and its dependencies. Computer simulations and physics-inspired models may help to understand the hidden aspects of such measures. They are low cost and noninvasive test opportunities to examine hypotheses that might not be ethically or practically applicable on a real human brain. With the help of simulations and computational models, it has become possible to explain the alterations in brain connectivity during progression of Alzheimer’s disease^33^, predict the optimal targets of deep brain stimulation^34,35^, explain epileptic seizures, find a patient-specific epileptogenicity of the brain regions to improve epilepsy surgeries at hospitals^36–39^, or develop novel treatments for Parkinson’s disease^40^. Well-studied physical models have been employed by neuroscientists to investigate different aspects of the brain. Among others, Ising model^41,42^, Potts model^43^, Kuramoto oscillators^44–46^ and FitzHugh-Nagumo oscillators^36,47–49^ have an extended proven record of assisting the scientists to unveil features and functions of the brain^50^.

In this work, we study the feasibility of modeling and simulating the mechanism behind empirical template flexibility patterns observed in previous studies^20,21^. We show that the flexibility pattern found in empirical functional magnetic resonance imaging (fMRI) data from 331 healthy participants performing an “(*N*-back) working memory” task^20,21^ can be reproduced using a model of coupled FitzHugh-Nagumo oscillators. FitzHugh-Nagumo model, although a simplified representation of a single neuron, is frequently employed as a general model for excitable media at a meso/macroscopic level^51,52^. The block design of our *N*-back task together with the large number of participants allows for an ideal investigation of module reconfiguration in functional network of the Brain. Our model integrates observed brain connectivity between regions of a widely accepted brain atlas with simple paradigmatic local neuronal dynamics of these regions. This approach allows for the gaining of insight while taking into account a realistic brain structure.

*Working memory* is widely defined as the cognitive system that is responsible for short-term retaining and manipulating of information in the brain in order to perform cognitive tasks^53–55^ in absence of external cues or prompts^56^. Amongst the current popular paradigms to measure working memory, the variants of *N*-*back* tasks first introduced by Kirchner^57^, play a central role. During these tasks, participants are required to observe a series of stimuli and respond when the same stimulus is presented as the one *N* trials back, where *N* can vary and is usually between 0 (control condition with the current trial) and 3. A meta-analysis of 24 studies done by Owen et al.^58^, on *N*-back associated brain regions, has found that the following brain areas are robustly activated: lateral premotor cortex, dorsal cingulate and medial premotor cortex, dorsolateral and ventrolateral prefrontal cortex, frontal poles, and medial and lateral posterior parietal cortex.

**Figure 1:**
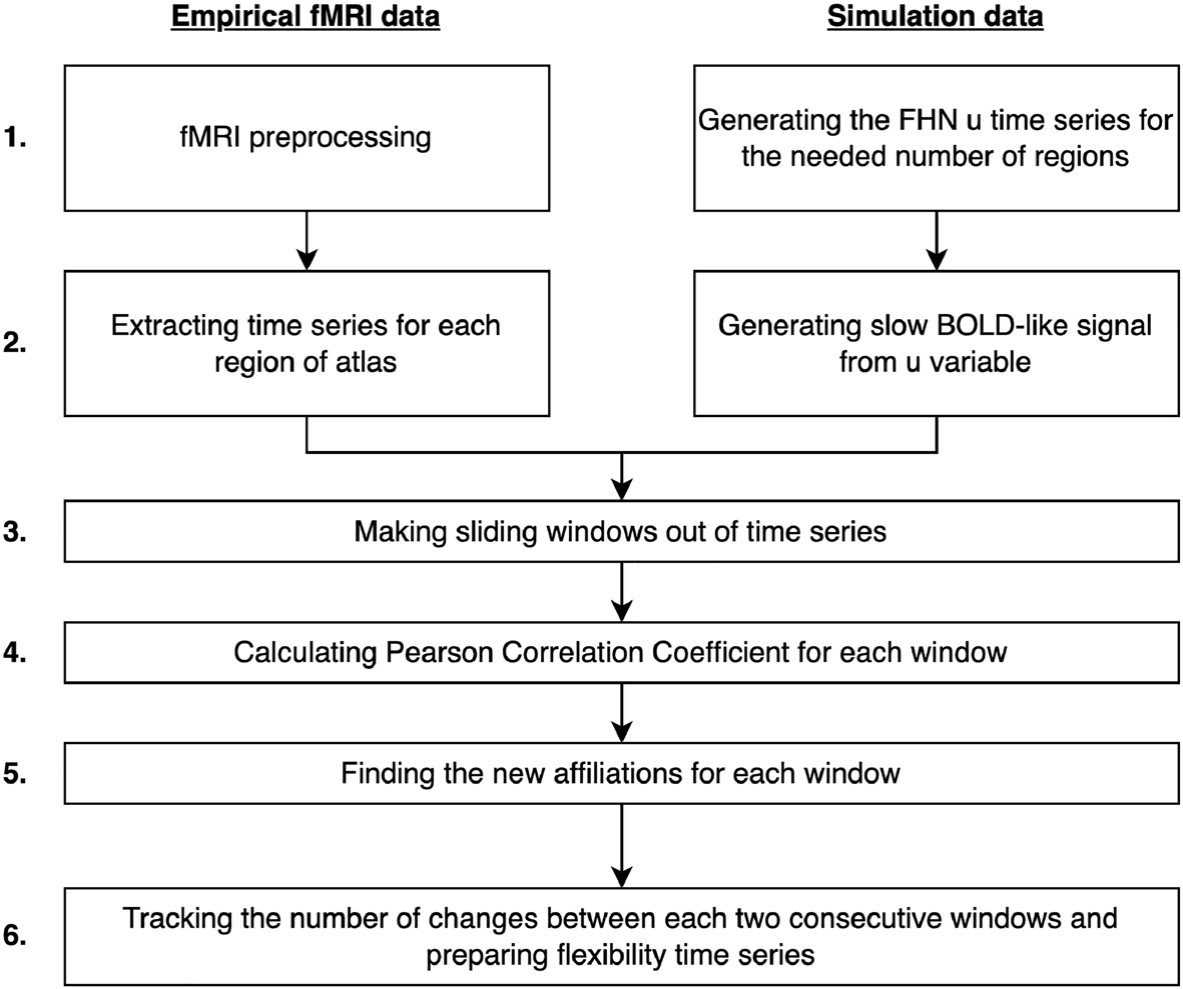
Empirical and simulated data pipelines. Empirical data from participants is collected and preprocessed. Time series for all brain regions are extracted. Pearson correlation coefficients between time series in every sliding window are calculated. The flexibility time series are generated based on the changes of node affiliations between consecutive windows. For the simulated data; time series are generated with the FitzHugh-Nagumo model and converted to slower oscillations via the Balloon-Windkessel model to resemble fMRI signals. The simulated time series are then treated like their empirical counterparts.

**Figure 2:**
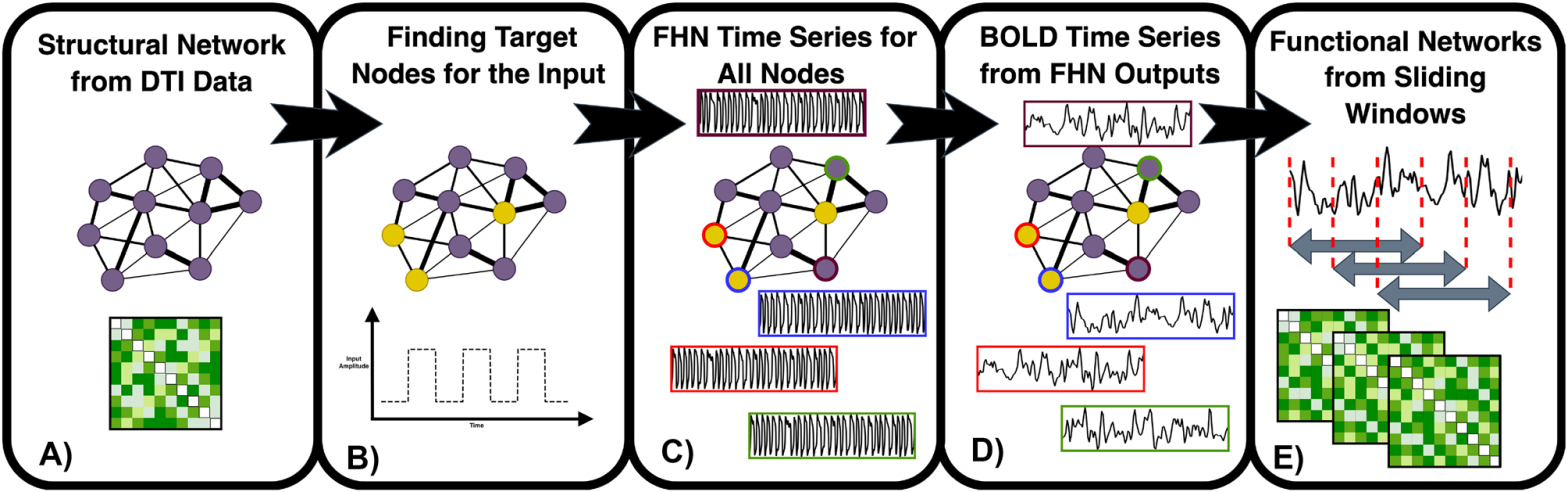
Schematic view of simulation steps. (**a**) Structural network is based on white matter Diffusion Tensor Imaging (DTI) data. (**b**) Nodes that receive the square wave input are marked and the magnitude of the input is decided. (**c**) FitzHugh-Nagumo time series are generated using the dynamics introduced in equation (1). (**d**) The time series of the *u*-variables of the FitzHugh-Nagumo model are passed to the Balloon-Windkessel model to produce slower Blood Oxygen Level Dependent (BOLD)-like signals. (**e**) The slow signals are treated like the empirical data. Sliding windows Pearson’s correlation coefficients are calculated between each pair of nodes and used as functional networks.

The empirical data in our study was derived from an N-back task that is designed in a blocked fashion. Each of the four blocks in our data set consisted of 30 seconds of the 0-back task followed by 30 seconds of the 2-back task. Participants were asked to either press the button corresponding to the number shown on the screen (0-back) or the number that was shown 2 steps ago (2-back). The recorded fMRI data of the 331 participants was then processed and a dynamical community measure of the nodes is extracted to study the flexibility in the grouping of brain regions while participants are alternating between 0 and 2-back blocks. For the modeling, we focus on reconstructing a similar flexibility pattern using a dynamical network of coupled FitzHugh-Nagumo (FHN) oscillators^36,46,59–63^. An averaged, high quality Diffusion Tensor Imaging (DTI) matrix serves as the weighted network structure^21^ for the coupled system. The blocks of the working memory tasks are modeled as square-wave inputs to the six brain regions associated with working memory. In order to compare the dynamics of the FitzHugh-Nagumo system with the empirical fMRI data, we use the Balloon-Windkessel hemodynamic model^64^ which allows for transforming neural activity to slow Blood Oxygen Level Dependent (BOLD) signals as measured by fMRI. The illustrations for the empirical and simulated procedures used are shown in Figs. 1 and 2.

In this study, we show the feasibility of modeling brain flexibility known from empirical brain data by a dynamical network approach. We introduce a simplified coarse-grained flexibility measure capturing the main patterns of known measures while being much faster to compute. We model the flexibility in a dynamical network of coupled oscillators, test for the importance of the brain’s network structure for flexibility and shed some light on the role of working memory associated brain regions in the observed flexibility.

This paper is organized as follows. In the ”Methods” section, details regarding our choice of empirical data, the simulation pipeline, the model of coupled FitzHugh-Nagumo oscillators, the choice of the input function and details on the flexibility measures calculations are presented. In the “Results” section, the findings in the three main domains of (1) reproducing the flexibility patterns and then investigating the effect of (2) structure and (3) the input node selection follow. Finally, the “Discussion” section wraps up the findings and their implications. There, we outline future research opportunities and limitations of our work.

## Methods

### Empirical data

The empirical data used in this study is taken from Chinichian et al.^21^. A multi-centric study at the Life and Brain Center of the University of Bonn, Charité—Universitätsmedizin Berlin, and Central Institute of Mental Health Mannheim with the approval by the Medical Ethics Committees of the three study sites and written informed consent of all participants was conducted. All fMRIs (functional Magnetic Resonance Imaging) were recorded by Siemens Trio 3T MRI scanner (Siemens Healthcare, Erlangen, Germany) with identical sequences. The working memory (WM) N-back task which is the focus on the current study was selected for a subset of 331 healthy participants^20,21^. Standard preprocessing including motion correction, slice-time correction, spatial smoothing with an FWHM of 9mm, high-pass temporal filtering with a 128s cutoff, and normalization to the Montreal Neurological Institute (MNI) template space with 3mm isotropic voxel size were carried out using SPM8^65^. Data collection and preprocessing are covered in greater detail by Esslinger et al.^66^. The working memory *N*-back task consisted of 30-second alternating blocks of 0-back followed by 2-back. As in both previous studies^20,21^ focusing on this data, a 15-volume window length with 14 volumes overlap was chosen for the sliding-window analysis, resulting in a total of 114 windows for each subject.

### Simulated data

The simulation in this study is aimed at generating artificial fMRI time series that show similar behaviour to those recorded from real human subjects who performed the working memory task used in the previous studies^20,21^. These artificial time series can then be investigated in detail to deepen our understanding of the phenomenon.

### FitzHugh-Nagumo model

To simulate the neural activity, we use a network of *N* FitzHugh-Nagumo (FHN) oscillators^60,67^, similar to the model studied by Ghosh et al.^61^. The FHN model serves as generic nonlinear oscillator model for the dynamics of each brain region, i.e., the nodes of the network. In this model, the activator variable $u_k$ and the inhibitor (or recovery) variable $w_k$ of each oscillator $k=1,\dotsc ,N$ follow the dynamics

1 $$\frac{\text{d}}{\text{d}t}\left(\begin{array}{l} \epsilon u_k \\ w_k \end{array}\right) = \left(\begin{array}{l} u_k - \frac{u_k^3}{3} - w_k + I_0 \\ u_k + a - b w_k \end{array} \right)- \sigma \sum _{l=1}^N g_{kl} \left(\begin{array}{l} u_l \\ 0 \end{array}\right) + \left(\begin{array}{l} I_{k}(t) \\ 0 \end{array}\right).$$

The parameters *a* and *b* are bifurcation parameters of the FitzHugh-Nagumo system, $I_0$ is the excitability parameter representing the common input given to each unit. The small parameter $\epsilon \ll 1$ sets the time-scale ratio of the fast activator and the slow inhibitor variable. In our model, each Brainnetome brain region (see Fig. 7, panel A) is seen as one single oscillator. The state variables $u_k$ and $w_k$ then describe the effective dynamics of that brain region. The coupling weight between oscillators *k* and *l* is given by the weighted adjacency matrix element $g_{kl}$ multiplied by the overall coupling strength coefficient $\sigma$.

Finally, $I_{k}(t)$, $k=1,\dotsc ,N$, is the external input which is given specifically to oscillator *k* at time *t*. The input is chosen based on the phenomenon under investigation. The model parameters together with their meanings are presented in Table 1.

The dynamics of the oscillators before they are coupled together is shown in Fig. S1 in Supplementary material. For a more detailed review on the behaviour of a FitzHugh-Nagumo oscillator, see Chapter 3 of Gerstner et al^67^ and the extensive dynamical visualizations with changing parameters by Izhikevich^68^.

**Table 1:** Parameters used in the FitzHugh-Nagumo model (1) and the Balloon-Windkessel model (3) to (10).

Symbol	Meaning
$\sigma = 1.8$	Overall coupling constant
$a=0.45, b=0.9$	Bifurcation parameters of the FitzHugh-Nagumo system
$I_0=0.8$	Excitability parameter
$\epsilon =0.1$	Controls time-scale separation between fast activation and slow inhibition
$c = 3$	Amplitude of the square-wave input $I_k$
$\tau _0 = 0.98$	Mean transit time of venous compartment (seconds)
$E_0 = 0.34$	Capillary bed net oxygen extraction fraction
$V_0 = 0.02$	Resting blood volume fraction
${\frac{1}{\alpha }} = 0.32$	flow-volume relationship power
$\tau _s = 0.65$	Time constant for signal decay (seconds)
$\tau _f = 0.41$	Time constant for auto-regulatory feedback from blood flow (seconds)

### Structural connectivity matrix G

Diffusion Tensor Imaging (DTI) is a magnetic resonance imaging (MRI) technique to obtain the map of neural tracts in the brain. This method uses the restricted diffusion of water in the tissue, an ellipsoid instead of a sphere, as a cue for the existence of a tract imposing some boundary conditions on the diffusion. The neural tracts information can serve as a structural network of the brain. It can assign a weight to each two spatial regions of the brain based on the tracts connecting them.

**Figure 3:**
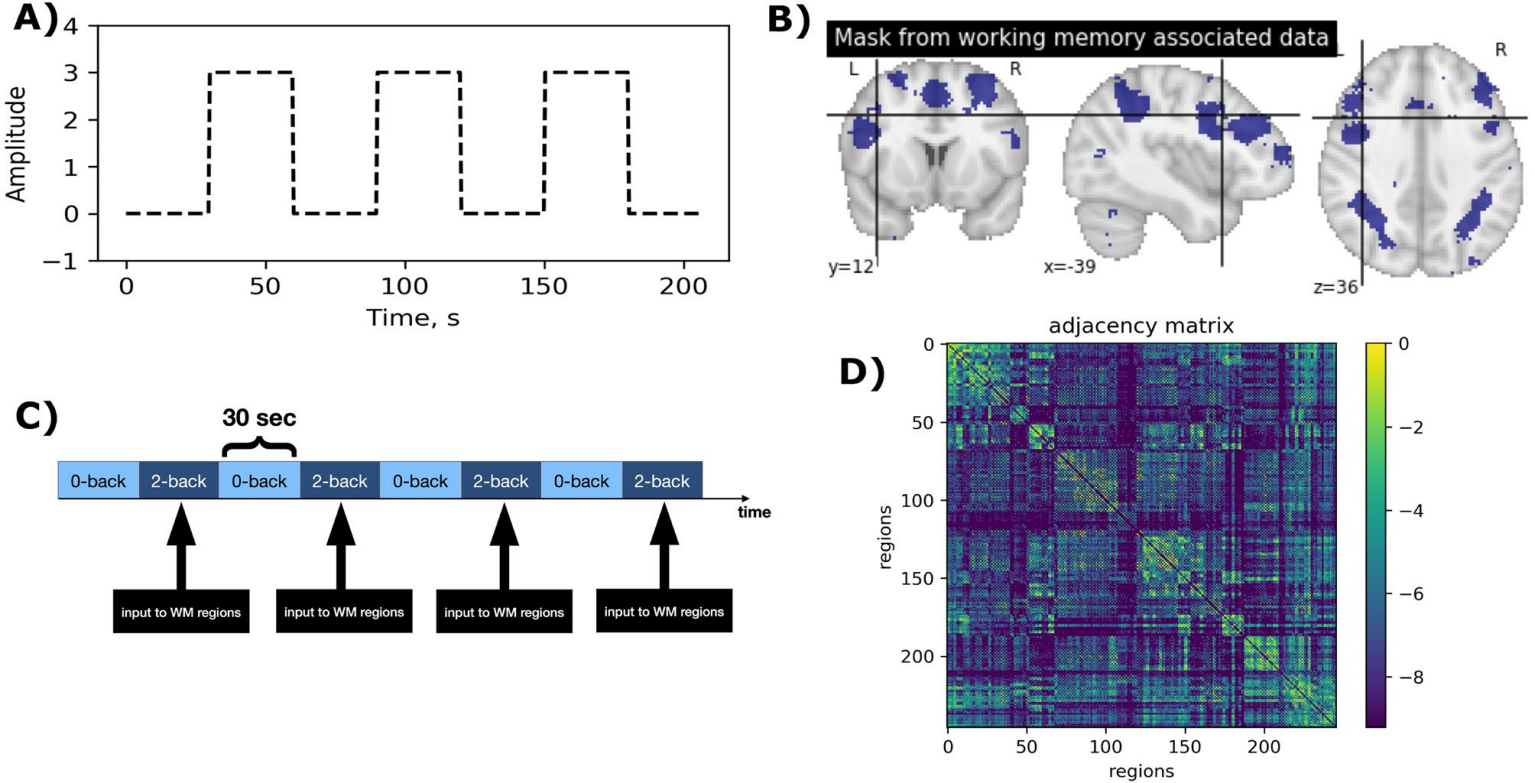
DTI matrix and input. (**A**) Shape of square-wave input given to the 6 selected nodes. (**B**) Working Memory associated areas extracted from Neurosynth engine^77^. The Brainnetome regions with bigger than 50% overlap are regions 25,26,29,63,127 and 211 (the full list of Brainnetome regions can be found in the supplementary material). (**C**) Alternating blocks of working memory task in *N*-back working memory task design. (**D**) Average DTI weighted adjacency matrix from 32 subjects of Human Connectome Project^70,78^ calculated by Horn et al. 2020 using Lead software^48–73^. For illustration purpose, $\textrm{log}(g_{ij}+10^{-4})$ is plotted^80^.

The structural connectivity weighted matrix G with elements $g_{ij}$ in Eq. (1) is expected to show the weights of connections between regions in the brain. For this purpose, a $246 \times 246$ averaged DTI matrix in Brainnetome atlas^69^ obtained from 32 adults was used(mean age 31.5 years ± 8.6 SD, 14 female^70^) who participated in Human Connectome Project (HCP)^71^ at Massachusetts General Hospital (“MGH HCP Adult Diffusion”). The averaged DTI was calculated using Lead group softwares^72,73^. The group’s connectome is accessible via LEAD-DBS portal^74^ and the processing steps are described by Horn et al.^75^. The weighted adjacency matrix G used in this paper (Fig. 3D) is multiplied by the overall coupling coefficient $\sigma$, which allows to tune the dynamics by a single parameter as in previous works^61,76^.

### Input

The *N*-back memory task in Chinichian et al.^21^ consists of 30-second blocks of 0-back followed by 30 seconds of 2-back. The 0-back condition serves as baseline in this task (see Fig. 3). The baseline includes all of the tasks, excluding the working memory component that is being measured in the experiment (such as viewing the slides, making decisions, and choosing). In other words, all biological processes other than the one that is the subject of the experiment should be present in the baseline condition. To mimic the block-design of our memory task in a simplified way, we use a square-wave input that can be written as:

2 $$\begin{aligned} I_k(t) = -c (2 \lfloor ft \rfloor -\lfloor 2ft \rfloor ), \end{aligned}$$

where $\lfloor x\rfloor$ is the floor function (i.e., the greatest integer less than or equal to *x*), *c* is a coefficient regulating the amplitude and $f={\frac{1}{T}}$ is the frequency with $T = 60$ s [for empirical reason of having 30-sec blocks for each condition]. An example input is shown in Fig. 3, panels A and C.

To select the regions that receive the input, we use a map of “working memory” associated areas from Neurosynth^81^. Neurosynth is a platform for large-scale, automated synthesis of functional magnetic resonance imaging (fMRI) data. A set of 1091 studies related to “Working Memory” are used to generate this association map. The areas are shown in Fig. 3. We calculated the overlap of Brainnetome regions with the Neurosynth map. The regions with most of their volume in working memory associated areas (over 50% overlap) are then selected as target regions to receive the input introduced in Eq. (2). In total, this procedure resulted in 6 target regions. These regions (with the numbering scheme of Brainnetome Atlas) are as follows:

- Regions 25 and 26: A6vl, ventrolateral area 6 from Middle Frontal Gyrus
- Region 29: A44d, dorsal area 44 from IFG from Inferior Frontal Gyrus
- Region 63: A6cvl, caudal ventrolateral area 6 from Precentral Gyrus
- Region 127: A7c, caudal area 7 from SPL from Superior Parietal Lobule
- Region 211: mAmyg, medial amygdala from Amygdala

### Balloon-Windkessel model

After numerically solving Eq. (1), the activator *u* of each FitzHugh-Nagumo oscillator is tranformed by the Balloon-Windkessel model^64^ to form a slower BOLD-like (Blood-oxygen-level-dependent) signal, which represents the measured signal during brain scans in the fMRI machines^82^. The Balloon-Windkessel model^64,83,84^ is a hemodynamic model that transforms synaptic activity into measured BOLD signals. This model uses the normalized venous volume *v*, the normalized total deoxyhemoglobin voxel content *q* and the resting net oxygen extraction fraction by the capillary bed $E_0$ to generate BOLD-like signal *y*(*t*) as follows:

3 $$\begin{aligned} y(t) \equiv V_0\left\{ k_1 \left[ 1-q(t)\right] + k_2\left[ 1-{\frac{q(t)}{v(t)}}\right] + k_3 \left[ 1-v(t) \right] \right\} , \end{aligned}$$

where $k_1 = 7E_0$ and $k_2 = 2$ and $k_3 = 2E_0 - 0.2$ and $V_0$ is the resting blood volume fraction. A list of parameters in Balloon-Windkessel model together with their meanings are summarized in Table 1

The dynamics of the volume *v* is given by:

4 $$\begin{aligned} {\dot{v}} = {\frac{1}{\tau _0}}\left[ f_{in} - f_{out}(v)\right] , \end{aligned}$$

which is dependent on the difference of the in-flow $f_{in}$ and the out-flow $f_{out}$ of venous compartment multiplied by a time constant $\tau _0$ that represents the average transit time (time to pass the venous compartment). The Windkessel model^84^ suggests that $f_{out}$ is dependent on the volume and can be written as

5 $$\begin{aligned} f_{out}(v) = v^{1/\alpha }, \end{aligned}$$

where $\alpha$, determined empirically, relates to the flow regime and the ratio of capacitance to compliance in balloon.

The dynamics of state variable *q* then reflects the difference of out-flow and in-flow of deoxyhemoglobin in the venous compartment

6 $$\begin{aligned} {\dot{q}} = {\frac{1}{\tau _0}}\left[ {\frac{E(f_{in},E_0)}{E_0} }- f_{out}(v){\frac{q}{v}}\right] \end{aligned}$$,

where $E(f_{in},E_0)$ shows the ratio of oxygen extracted from the inflow to the delivered amount and is assumed to depend on oxygen arriving with the in-flow:

7 $$\begin{aligned} E(f_{in},E_0)= 1 - (1-E_0)^{\frac{1}{f_{in}}}. \end{aligned}$$

**Figure 4:**
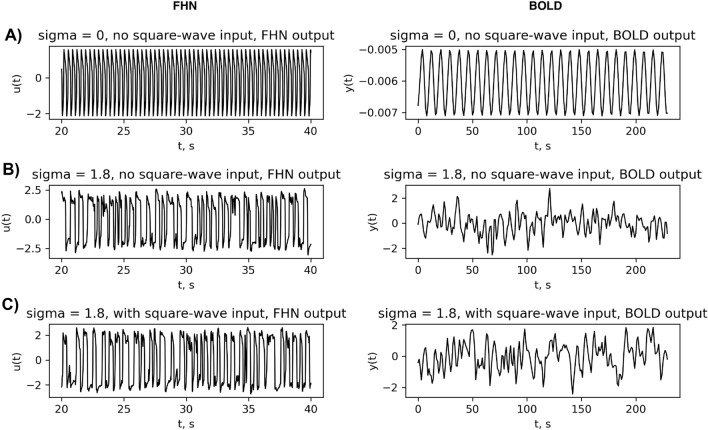
FitzHugh-Nagumo (FHN) and Balloon-Windkessel models outputs. An example plot for the outputs of FHN and Balloon-Windkessel models for a region in the middle of the sorted list of weighted connections [Region 148 from Brainnetome] region for the 3 cases; Top: $\sigma = 0$ and no square-wave input to any region, $I_k\equiv 0$ for all $k=1,\dotsc ,N$. Middle: $\sigma = 1.8$ and no square-wave input to any region. Bottom: $\sigma = 1.8$ and square-wave input $I_k(t)$ (see Eq. (2)) given to the 6 selected working memory regions. See supplementary material Fig. S8 for the least connected node (115), Fig. S9 most connected node (230) and Fig. S10 a node that is directly receiving input (63).

The in-flow, $f_{in}$ changes based on the induced signal that depends on the normalized neuronal activity ${\hat{u}}_k(t)$, which is the activator variable of FitzHugh-Nagumo oscillators in our study:

8 $$\begin{aligned} {\hat{u}}_k(t) = {\frac{u_k(t)-{\overline{u}}_k}{SD(u_k(t))}}, \end{aligned}$$

where ${\overline{u}}_k$ is the average value of time series ${u}_k(t)$ over the whole *t* span and SD indicates the standard deviation of the time series. Furthermore,

9 $$\begin{aligned}&{\dot{f}}_{in} = s \end{aligned}$$

10 $$\begin{aligned}&{\dot{s}} = \epsilon _B {\hat{u}}_k(t) - {\frac{s}{\tau _s}} - {\frac{f_{in}-1}{\tau _f}}, \end{aligned}$$

where $\epsilon _B$ is the efficiency with which neuronal activity causes an increase in signal,$\tau _s$ is the time constant for signal decay and $\tau _f$ is time constant for auto-regulatory feedback from blood flow. Solving the Eqs. (4), (6), (9), and (10) we are able to calculate the desired *y*(*t*) which has the nature of slower BOLD (Blood-oxygen-level-dependent) signals we need for the intercomparison with empirical data (see Fig. 4 for examples of FitzHugh-Nagumo vs Balloon-Windkessel outputs). The parameters used for the simulation are presented in Table 1.

### Summary of simulation steps

The FitzHugh-Nagumo time series with parameters given in Table 1 and the time-dependent input introduced in Eq. (2) given to regions introduced in the ”Input” subsection above are generated on the nodes of weighted connectivity matrix G. The z-score normalized (or standardized) output is then fed to Balloon-Windkessel model introduced in subsection “Balloon-Windkessel model” and the result is treated as a single subject fMRI brain signals. The simulation is repeated 300 times with different random initial conditions to account for the effect of fluctuations due to random initial conditions.

### Calculating template flexibility for empirical and simulated fMRI time series

We obtain the elements $a_{ij}^{win}$ of the functional adjacency matrix $A^{win}$ for time window *win* from the Pearson correlation coefficients as follows:

11 $$\begin{aligned} a_{ij}^{win} = {\frac{{\sum _{k=1}^{l}\left( y_i(t_k^{win}) - \mu ^{win}_i \right) (y_j(t_k^{win}) - \mu ^{win}_j)}}{{\sqrt{\sum _{k=1}^{l}(y_i(t_k^{win}) - \mu ^{win}_i)^2}\sqrt{\sum _{k=1}^{l}(y_j(t_k^{win}) - \mu ^{win}_j)^2}}}}, \end{aligned}$$

where the sums are taken over the $l=15$ time points of the sliding windows *win* with temporal length $T=l\Delta t$ and $\Delta t=2$ s. Here, $\mu ^{win}_i = \frac{1}{l}\Sigma _{k=1}^{l}y_i(t_k^{win})$ is the average and $t_k^{win} = {win}\cdot T + k\Delta t$ are the absolute times of window *win*. The template module matrix M for the template flexibility measure is based on the overlap of Findlab networks^85^ and the 246 Brainnetome atlas regions^69^ as listed in Table 3 in the Supplementary material similar to Chinichian et al.^21^. Matrix M is of the size $246 \times 15$ where 246 is the number of brain regions [nodes, oscillators] and 15 is the number of a-priori modules. The element $m_{ij}$ of matrix M equals to 1 if region *i* belongs to module *j* in the template and 0 otherwise. Note that this binary affiliation is a level of simplification. In practice, the overlaps could be quantified to range between 0 and 1. Next, we calculate the weights each node has in connecting to every a-priori module with matrix $H^{\prime }$:

12 $$\begin{aligned} H ^{\prime } = |A^{win}| \times M. \end{aligned}$$

Here, $|A^{win}|$ is the matrix whose elements are the absolute values $|a^{win}_{ij}|$ and $\times$ denotes external matrix multiplication. We then normalize every element $h ^{\prime }_{i,j}$ of the $(246\times 15)$-matrix $H ^{\prime }$ to the size $K_j$ of the *j*-th module in the template (i.e. $K_j=\sum _{i=1}^{246} M_{ij}$ is the column sum associated to module *j* in matrix *M*) and call the resulting matrix *H* with elements $h_{ij}$:

13 $$\begin{aligned} h_{ij} = {\frac{h^{\prime }_{ij}}{K_j}}. \end{aligned}$$

With *H* we have calculated the strength of affiliations to each module for all nodes. The strongest module affiliation per node is selected as the winner, forming an “affiliation vector” $\mathbf {\Omega }_{win}$ for each time window *win*:

14 $$\begin{aligned} \mathbf {\Omega }_{win} = \begin{bmatrix} \mathop {\mathrm {arg\,max}}\limits _j(H_{1j}) \\ \vdots \\ \mathop {\mathrm {arg\,max}}\limits _j(H_{246 j}) \\ \end{bmatrix} \end{aligned}$$

where $\mathop {\mathrm {arg\,max}}\limits _j(H_{ij})$ delivers the label of the module with which node *i* has the largest affiliation.

Finally, the flexibility value between two consecutive windows is calculated by

15 $$\begin{aligned} F^{(win,win-1)} = 1-{\frac{1}{246}}\sum _{i=1}^{246} \delta _{\omega _i^{win},\omega _i^{win-1}}, \end{aligned}$$

where $\omega _i$ denotes the *ith* element of $\mathbf {\Omega }_{win}$ and the Kronecker delta $\delta _{\omega _i^{s},\omega _j^t}$ is 1 if $\omega _i^s=\omega _j^t$ and 0 otherwise.

### Distance flexibility of consecutive windows weighted adjacency matrices

In addition to the template flexibility measure in Eq. (15), we define a less complex measure of reconfiguration in the brain. We use this measure in parallel to the template flexibility to compare the empirical and simulated data and call it **“Distance Flexibility”** or *d*-measure. This measure is independent of our choice of template modules. To calculate *d*, the same procedure as for template flexibility is followed until the 4th step in the previous section in Fig. 1, where we have all the weighted adjacency matrices $A^{win}$. Then, the Pearson distance between any two consecutive windows $win-1$ and *win* is defined as 1 minus the Pearson correlation coefficient:

16 $$\begin{aligned} \begin{aligned} \small d^{(win,win-1)} = 1-{\frac{\sum _{i=1}^{N}\sum _{j=1}^{N}\left( A^{win}_{ij} - {\bar{A}}^{win}\right) \left( A^{(win-1)}_{ij} - {\bar{A}}^{(win-1)}\right) }{\sqrt{\sum _{i,j=1}^{N}(A^{win}_{ij} - {\bar{A}}^{win})^2}\sqrt{\sum _{i,j=1}^{N}\left( A^{(win-1)}_{ij} - {\bar{A}}^{(win-1)}\right) ^2}}}, \end{aligned} \end{aligned}$$

where $N = 246$ is the number of nodes and ${\bar{A}}^{win}$ is the average of the elements of $A^{win}$:

17 $$\begin{aligned} {\bar{A}}^{win}={\frac{1}{N^2}}\sum _{i,j=1}^{N}a^{win}_{ij}. \end{aligned}$$

A time series $d^{win,win-1}$, of *d*-measures can be calculated from all pairs of consecutive windows. In other words, we first use the Pearson correlation to obtain $A^{win}$ from the brain activity or the simulated signals. After that, we use the Pearson distance again, this time between elements of different $A^{win}$, to obtain the distance flexibility (*d*-measure). The *d*-measure is closely related to the template flexibility because if the two consecutive weighted adjacency matrices are very similar, the correlation is high. Note that it does not necessarily hold the other way round, very different matrices (by element values) can still have large correlation coefficients. On the other hand, when two consecutive weighted adjacency matrices are dissimilar, the chance of switching is high and in many cases the correlation coefficient is also small. There are mathematical cases that the two measures are not showing similar patterns (see Fig. 6 as an example of the measures deviating) but they are defined in a related way. Despite the easy calculation and intuitive meaning, distance flexibility is not based on capturing the node affiliations and changes in them, it is a global value that quantifies dissimilarities between functional weighted adjacency matrices in different windows.

### Generating $G^\prime$ by shuffling *G* elements

To generate the randomized weighted adjacency matrix $G^\prime$ which is needed for our structural investigation, the elements of the upper triangle in the original weighted adjacency matrix *G* are randomly shuffled and then used to fill the lower triangle such that the matrix is symmetric and thus preserves the undirectedness of the graph.

### Node selection scenarios

Figure 7B shows the weighted degree distribution of the DTI matrix. The six working memory nodes found on the empirical data by the method described in the ”Input” subsection above, fall mainly in the middle part of the node degree histogram. See the complete list of node indices in supplementary material. We test the importance of weighted degree of stimulated nodes (also known as the “strength” of nodes^7^ ) through the three distinct scenarios that follow:

1. Stimulating the first 6 nodes with lightest sum of weighted connections. (Light scenario with weakest nodes)
2. Stimulating 6 nodes around the median of weighted connections sums. (Mid scenario with median strength nodes)
3. Stimulating the last 6 nodes with heaviest sum of weighted connections. (Heavy scenario with strongest nodes)

The 6 nodes for all scenarios are shown in bold on the list in the supplementary material along with the full list of Brainnetome regions in Table 2 in the Supplementary Material

## Results

### Reproducing template flexibility pattern

In this section, we address the question whether a simulation based on a FitzHugh-Nagumo oscillator model with empirical network connectivity can reproduce patterns of template flexibility that are observed in the empirical fMRI data. The computation of the template flexibility time series for simulated data is analogous to the corresponding computation for the empirical data (Fig. 1).

**Figure 5:**
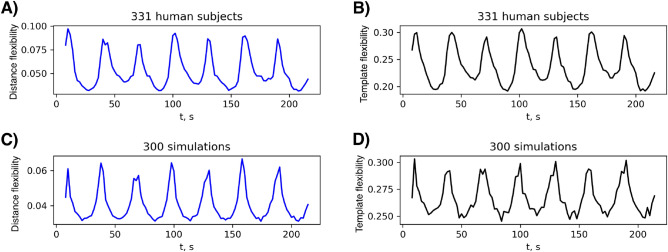
Comparing empirical and simulated cases. Distance flexibilities on the left column and template flexibilities on the right column in empirical data (first row, panels **A** and **B**) and simulated data (second row, panels **C** and **D**). Pearson correlation coefficient for the two template flexibility time series is 0.85 and for the two distance flexibility time series is 0.87 (with mean absolute error (MAE) of 0.01 and 0.03 respectively). A ”subject” in empirical data means one participant and a ”simulation” in simulated data means a single run with a random initial condition [empirical data: averaged over 331 participants and simulated data averaged over 300 random initial conditions]. Each sliding time window is covering 30 s and two consecutive time windows have 28 s overlap.

The time series of the a-priori template flexibility (see Eq. (15) and Chinichian et al.^21^) is computed for empirical and simulated data (Fig. 5 panels B and D). Further, for the same data the corresponding simplified distance flexibility measure of time series of functional matrices, as discussed above and defined in Eq. (16) in “Methods”, is shown (Fig. 5 panels A and C). The averaged (over different initial conditions) simulated data shows a similar pattern to the averaged (over different participants) empirical data. The Pearson correlation coefficient for the two template flexibility time series shown in Fig. 5 equals 0.85 and for the two distance flexibility time series 0.87 (with mean absolute error of 0.01 and 0.03 respectively).

During the performance of the task in each block, the brain is assumed to stay in a specific configuration which changes when moving to the next block. To account for effects occurring due to specific initial conditions, the simulation is repeated for 300 times with the same parameters and different random initial conditions and then averaged across all runs. Similarly, the empirical data is smoothed as a result of averaging across 331 participants.

### Effect of structure

**Figure 6:**
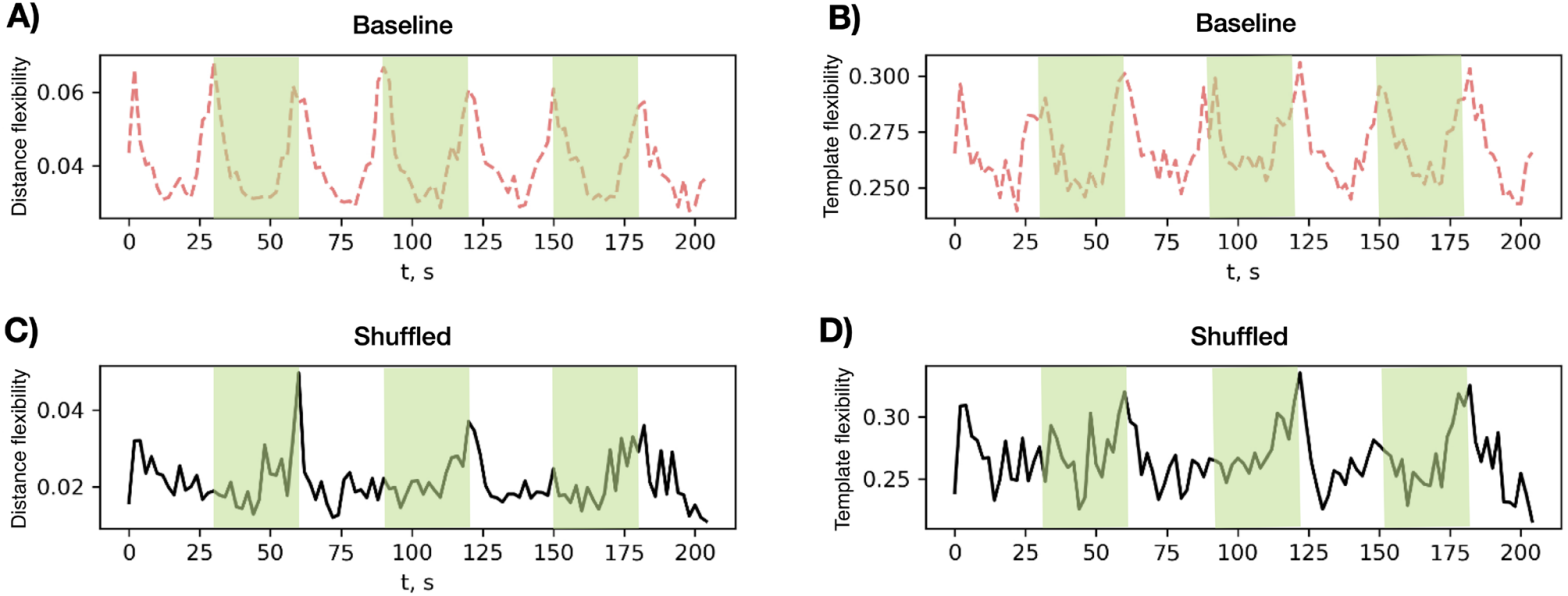
Simulation with randomly shuffled connectivity matrix. Comparison of two ensembles of 50 simulations each with different initial conditions on the empirical DTI-based connectivity matrix *G* (”Baseline”, panels **A** and **B**) and a randomly shuffled version $G^\prime$ with the same weight distribution (“Shuffled”, panels **C** and **D**). All other simulation parameters except the structural matrix were kept the same (equal to those in Fig. 5). Green stripes show the time windows with the external input. Left column compares the distance flexibility values and right column the template flexibility outcomes. The Pearson correlation coefficient for the two template flexibility time series is 0.64 and for the two distance flexibility time series is 0.42 (with mean absolute error (MAE) of 0.01 for both cases).

The simulations on the randomly shuffled network do not show the same strongly regular oscillatory patterns for the distance and the template flexibility measures as those that are seen in the empirical DTI based simulation baseline case (see Fig. 6). The Pearson correlation coefficient between the two template flexibility time series shown in Fig. 6 is 0.64 and between the two distance flexibility time series is 0.42 (with mean absolute error MAE of 0.01). Both cases (baseline and shuffled DTI) were computed with the same ensemble size of 50 runs with different random initial conditions. The result suggests that the specific connectivity is an inseparable precondition for the flexibility patterns observed in the brain. The disappearance of the regular oscillatory pattern in the shuffled DTI ensemble shows that the regular input (the square wave) alone is not sufficient to induce the observed oscillations. We note at this point that the analysis presented in this section is intended to showcase the possible perspective for our proposed modeling approach. With increasing the number of initial conditions to 300, the shuffled version converges to a more regular pattern, although on the smaller number of initial conditions, the baseline converges already to the regular pattern while the shuffled version stays irregular. See Supplementary material figure S4 for further simulation. A deeper analysis might allow for strong conclusions on the importance of the particular topological brain structure for the brain’s flexibility.

### Effect of node selection

In this section, we ask as to what extent the selection of nodes that receive input is important for the flexibility patterns. For this question, we investigate flexibility patterns when the input in Eq. (2) is given to regions other than those marked as associated with the working memory. We test three scenarios: (1) stimulating the least connected nodes (light scenario), (2) stimulating the middle (medium) connected nodes (mid scenario), (3) stimulating the best connected nodes (heavy scenario).

**Figure 7:**
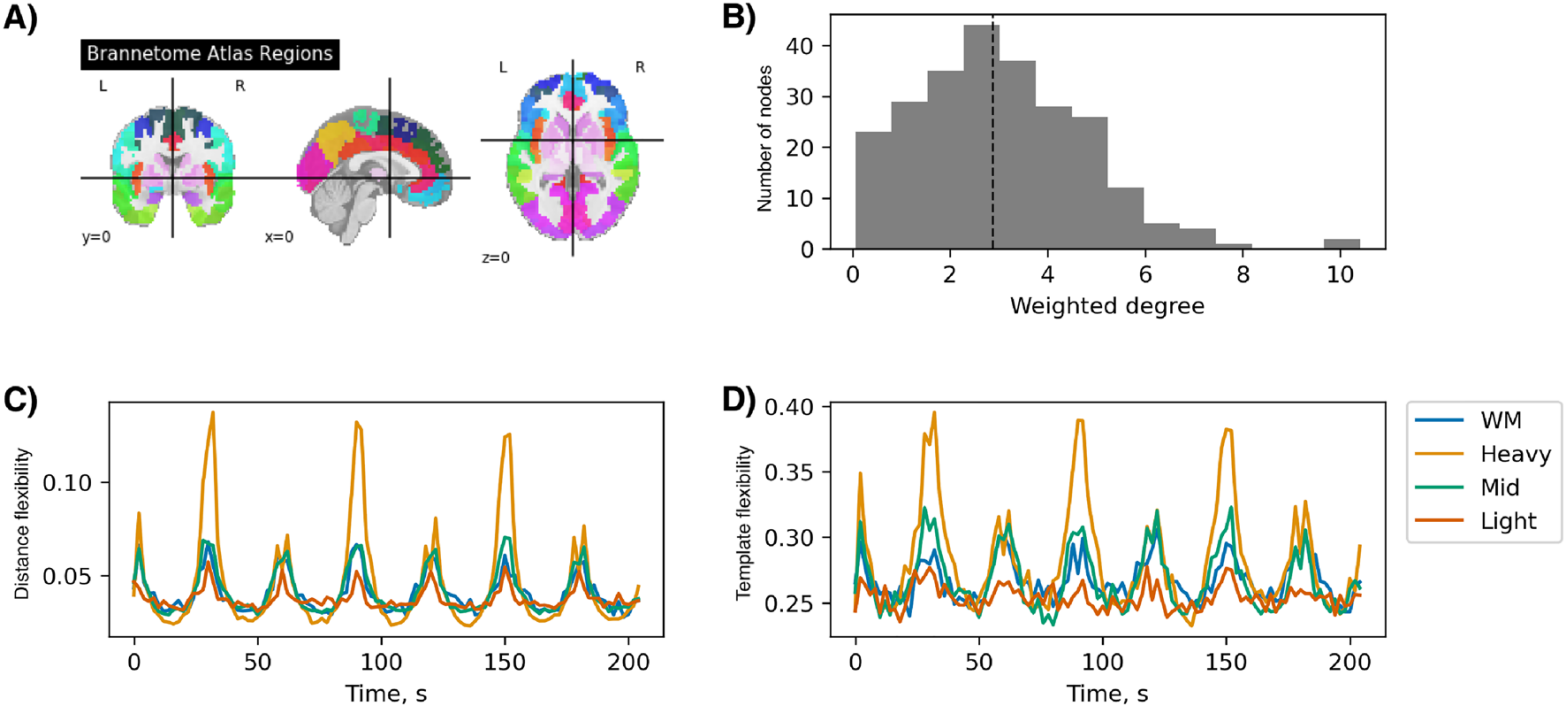
Node selection scenarios/ Influence of stimulated brain region on flexibility outcome. (**A**) Visualization of Brainnetome atlas regions. (**B**) Histogram of weighted degrees for all 246 nodes in the DTI matrix. The dashed line shows the median value of weighted degrees. (**C**) Comparison between distance flexibility measure for the 4 simulation scenarios of WM (nodes associated empirically to working memory. See “Node selection scenarios” subsection for more information), Heavy (heavy scenario, referring to the weighted node degree), Middle (mid scenario) and Light (light scenario) nodes being stimulated by $I_k(t)$. In oragne-colored Heavy scenario, the symmetry between the start of input vs no-input blocks is broken. (**D**) Comparison of template flexibility time series for the 4 scenarios.

The results of our three simulation scenarios together with the original working memory (WM) case are presented in Fig. 7C, D. Exemplary time series of the activator variable *u* from the FitzHugh-Nagumo model for the three scenarios are also shown in Figs. S5, S6 and S7 in the Supplementary Material.

The light scenario shows a visibly weaker oscillatory pattern. This could be explained by the fact that the lightest nodes are not well connected to the rest of the network and the input would not strongly affect the neighboring nodes through the couplings. The mid scenario has the most similar pattern to the working memory area. Indeed, checking the distribution of working memory nodes in the weighted degree list (see Supplementary Material), the mid scenario is the closest to it. The most notable result of this simulation concerns the heavy scenario, when the input is given to the heavily connected nodes. In this case the symmetry between the blocks’ valleys (no working memory input) and peaks (working memory input) is broken and the start of 2-back task (peak part) is marked by deeper valleys, and a larger template flexibility is observed for the 2-back reconfiguration. In the empirical data, marginally higher flexibility was observed during the 2-back compared to 0-back blocks (*t* = $- 2.9$, $p = 0.03$ [*t* and *p* refer to *t*-value and *p*-value from statistical hypothesis testing] ). This might suggest that the working memory regions are empirically well-connected but they are not the main weight hubs of the brain network.

## Discussion

This study shows that a simple dynamical model of coupled neural populations with an alternating external input exhibits flexibility patterns akin to those observed in empirical fMRI scans of subjects performing working memory tasks. Our model enables us to study the impact of structural aspects (such as the specific connectivity and the stimulated brain regions) on the simulated flexibility. The structure of the brain network plays a significant role in the cooperation of brain areas and the execution of cognitive tasks. We find evidence that shuffling the link weights in the model causes the previously observed similarities to disappear, indicating a strong influence of the connectivity on the flexibility dynamics. This finding aligns well with several previous studies that highlight the importance of brain structure in cognitive functions^32,86–89^.

Additionally, the regions stimulated during the execution of a cognitive task influence the patterns. Most notably, the strength of the observed oscillations in flexibility between 2-back and 0-back seems to depend on the total weighted degree (strength) of the stimulated nodes. Stimulating nodes with a median strength leads to the outcomes most similar to the empirically relevant nodes. On the other hand, the precise location of the stimulated nodes, or the overlap of their associated brain atlas regions with the brain areas that are empirically related to working memory does not influence the outcome much. Interestingly, when highly interconnected nodes are stimulated, the difference in maximum flexibility between task blocks with and without input increases. This is matched by the small but statistically significant difference between the flexibility peaks in the empirical data. These results suggest that the cognitive tasks which involve the contribution of weight hubs in the network can significantly influence the regrouping behaviors of the nodes in the brain. This discovery holds significance as it can provide valuable insights into the investigations regarding the link between cognitive task performance, network hubs, and brain disorders^90–92^.

We believe that a great value of our simplified model is that it facilitates the discovery of such effects by allowing for the variation of model parameters. Since the flexibility difference disappears when less interconnected nodes are stimulated, our model furthermore suggests an explanation for that effect, which future research will have to substantiate: namely, that working memory associated brain areas are well connected nodes of the network and The more heavy the nodes linked to a task are, the more extensive adjustments are required in brain modules to execute the task.

In conclusion, the observed flexibility pattern in the brain is the result of a complex interaction between the structure of the brain and the activation of cognitively relevant regions during the execution of each task. A short report of the capacity of template flexibility measure to classify schizophrenia patients performing a “theory of mind” task can be found here^32^. Future studies that include modifying the model’s structural and functional components in accordance with the literature on schizophrenia might help in replicating and explaining the larger variation in patients compared to the control group. Despite the success in reconstructing the flexibility pattern, our model has severe simplifications and limitations. Part of this comes from the *N*-back task nature. Although the *N*-back task is a well-studied manipulation of working-memory, the task’s structure makes it difficult to differentiate the cognitive processes of working-memory maintenance from information manipulation^20,93^. While using the averaged DTI-based matrix has clear benefits, it also has drawbacks; (1) diffusion MRI is a powerful technique but can only provide an estimation of white matter pathways organisation^94^, (2) tensor-based methods consistently underestimate the connectivity patterns because of white matter complex geometry (e.g. crossing fibers)^95^, and (3) the number of streamlines estimated with tractography is only a poor approximation of the actual microscopical fibre count and should be used with caution^96^. Our study was simplified by taking 0-back as the baseline and associating input only with the 2-back condition blocks. Although this follows the idea of task design (stimulating all elements of cognition in the baseline condition except for the aspect being researched), it is a very rough approximation of the cognitive process. Another limitation is the ability of a single Fitzhugh-Nagumo oscillator to simulate the overall neural activity of each brain area as large as a few cubic centimeters. The choice of region size has a substantial impact on our spatial resolution. Therefore, we neglect the critical fine-scale diversity in the complexity of human brain function. Yet even with all simplifications and limitations, we believe that this study sheds some light on the possibility of modeling brain reconfiguration and the significance of brain structure in the reconfiguration process.

### Supplementary Information

Supplementary Information.

## Data Availability

The averaged flexibility empirical time serie in this study was taken from^21^ and could be requested from the corresponding author, access to individual subject’s fMRI time series for any further analysis requires special permission from the imaging project PIs and needs to be justified. Please see^21,97,98^ for more information on corresponding fMRI projects (IntegraMent and MooDS) and their details. The computer simulated flexibility time serie can be generated using the open access GitHub codes of the project. Please contact the corresponding author to address any issue regarding the codes.
