# Supplementary material for: Modeling brain network flexibility in networks of coupled oscillators: a feasibility study
Source: Sci Rep. 2024 Mar 8;14:5713. doi: 10.1038/s41598-024-55753-8 (PMC10923875; doi:10.1038/s41598-024-55753-8)
Supplement: Supplementary file 1 — Supplementary Information. [file 41598_2024_55753_MOESM1_ESM.pdf]

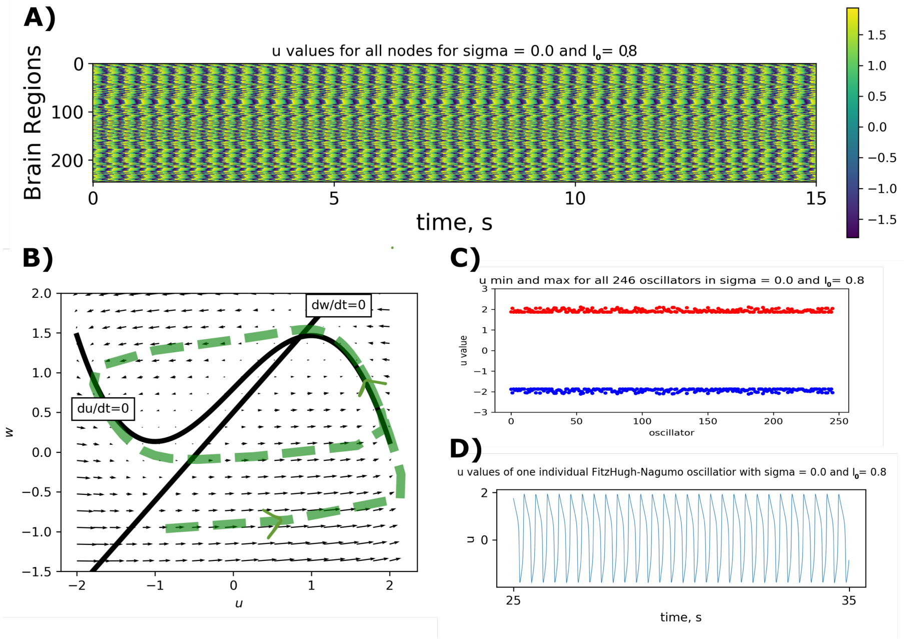

Figure S1: **Oscillator dynamics when there is no coupling ($\sigma = 0$)**
A) Space-time plot of all 246 oscillators, B) Phase portrait of one oscillator. C) minimum and maximum of u time series for all oscillators. Blue dots are minima and red dots are maxima, D) Oscillation of one single oscillator for 10 seconds. Panels A and C show the oscillations of all the oscillators. With no coupling to connect the oscillators, each unit is only different in its initial condition. Panel B shows the nullclines and a trajectory of one single oscillator. Panel D shows the u time series for a longer period of time. Parameters b and a help tuning the system for the simple case of one intersection point between the two nullclines and the move of intersection on the peak or trough of the u-nullcline based on the respective case.

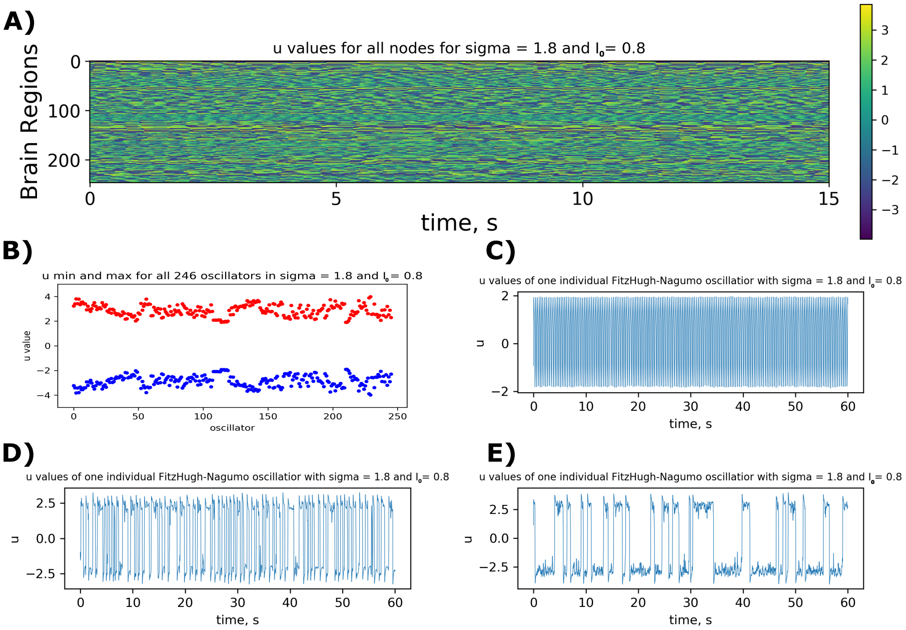

Figure S2: **Oscillator dynamics with $\sigma = 1.8$, $I_0 = 0.8$ and without square-wave input, i.e.** $I_k = 0\ \forall k$. A) Space-time plot (u_k vs. t) of all 246 oscillators, B) minimum and maximum values of $u_k(t)$ for all oscillators, C), D) and E) $u_k(t)$ for the node with the lowest, median and highest weighted degree.

Table T1: Number and Name of modules based on Findlab networks [**801550:19378567**]. Reused from [**chinichian2023fast**]

Module Number (Short Name)	Module Name
Module 1 (M1)	Anterior Salience
Module 2 (M2)	Auditory
Module 3 (M3)	Basal Ganglia
Module 4 (M4)	Dorsal Default Mode Network (dDMN)
Module 5 (M5)	High Visual
Module 6 (M6)	Language
Module 7 (M7)	Left Executive Control (LECN)
Module 8 (M8)	Posterior Salience
Module 9 (M9)	Precuneus
Module 10 (M10)	Primary Visual
Module 11 (M11)	Right Executive Control (RECN)
Module 12 (M12)	Sensorimotor
Module 13 (M13)	Ventral Default Mode Network (vDMN)
Module 14 (M14)	Task Positive
Module 15 (M15)	Undefined (untagged nodes listed in **??**)

Table T2: **Brainnetome atlas regions**

Table reused from [**801550:19378967**, **chinichian2023fast**]

Lobe	Gyrus	Label ID.L	Label ID.R	
Frontal Lobe	SFG, Superior Frontal Gyrus	1	2	A8m, medial area 8
		3	4	A8dl, dorsolateral area 8
		5	6	A9l, lateral area 9
		7	8	A6dl, dorsolateral area 6
		9	10	A6m, medial area 6
		11	12	A9m,medial area 9
		13	14	A10m, medial area 10
	MFG, Middle Frontal Gyrus	15	16	A9/46d, dorsal area 9/46
		17	18	IFJ, inferior frontal junction
		19	20	A46, area 46
		21	22	A9/46v, ventral area 9/46
		23	24	A8vl, ventrolateral area 8
		25	26	A6vl, ventrolateral area 6
		27	28	A10l, lateral area10
	IFG, Inferior Frontal Gyrus	29	30	A44d,dorsal area 44
		31	32	IFS, inferior frontal sulcus
		33	34	A45c, caudal area 45
		35	36	A45r, rostral area 45
		37	38	A44op, opercular area 44
		39	40	A44v, ventral area 44
	OrG, Orbital Gyrus	41	42	A14m, medial area 14
		43	44	A12/47o, orbital area 12/47
		45	46	A11l, lateral area 11
		47	48	A11m, medial area 11
		49	50	A13, area 13
		51	52	A12/47l, lateral area 12/47
	PrG, Precentral Gyrus	53	54	A4hf, area 4(head and face region)
		55	56	A6cdl, caudal dorsolateral area 6
		57	58	A4ul, area 4(upper limb region)
		59	60	A4t, area 4(trunk region)
		61	62	A4tl, area 4(tongue and larynx region)
		63	64	A6cvl, caudal ventrolateral area 6
	PCL, Paracentral Lobule	65	66	A1/2/3ll, area1/2/3 (lower limb region)
		67	68	A4ll, area 4, (lower limb region)
Temporal Lobe	STG, Superior Temporal Gyrus	69	70	A38m, medial area 38
		71	72	A41/42, area 41/42
		73	74	TE1.0 and TE1.2
		75	76	A22c, caudal area 22
		77	78	A38l, lateral area 38
		79	80	A22r, rostral area 22
	MTG, Middle Temporal Gyrus	81	82	A21c, caudal area 21
		83	84	A21r, rostral area 21
		85	86	A37dl, dorsolateral area37
		87	88	aSTS, anterior superior temporal sulcus
	ITG, Inferior Temporal Gyrus	89	90	A20iv, intermediate ventral area 20
		91	92	A37elv, extreme lateroventral area37
		93	94	A20r, rostral area 20
		95	96	A20il, intermediate lateral area 20
		97	98	A37vl, ventrolateral area 37
		99	100	A20cl, caudolateral of area 20
		101	102	A20cv, caudoventral of area 20
	FuG, Fusiform Gyrus	103	104	A20rv, rostroventral area 20
		105	106	A37mv, medioventral area37
		107	108	A37lv, lateroventral area37
	PhG, Parahippocampal Gyrus	109	110	A35/36r, rostral area 35/36
		111	112	A35/36c, caudal area 35/36
		113	114	TL, area TL (lateral PPHC, posterior parahippocampal gyrus)
		115	116	A28/34, area 28/34 (EC, entorhinal cortex)
		117	118	TI, area TI(temporal agranular insular cortex)
		119	120	TH, area TH (medial PPHC)
	pSTS, posterior Superior Temporal Sulcus	121	122	rpSTS, rostroposterior superior temporal sulcus
		123	124	cpSTS, caudoposterior superior temporal sulcus

Parietal Lobe	SPL, Superior Parietal Lobule	125	126	A7r, rostral area 7
		127	128	A7c, caudal area 7
		129	130	A5l, lateral area 5
		131	132	A7pc, postcentral area 7
		133	134	A7ip, intraparietal area 7(hIP3)
	IPL, Inferior Parietal Lobule	135	136	A39c, caudal area 39(PGp)
		137	138	A39rd, rostrodorsal area 39(Hip3)
		139	140	A40rd, rostrodorsal area 40(PFt)
		141	142	A40c, caudal area 40(PFm)
		143	144	A39rv, rostroventral area 39(PGa)
		145	146	A40rv, rostroventral area 40(PFop)
	Pcun, Precuneus	147	148	A7m, medial area 7(PEp)
		149	150	A5m, medial area 5(PEm)
		151	152	dmPOS, dorsomedial parietooccipital sulcus(PEr)
		153	154	A31, area 31 (Lc1)
	PoG, Postcentral Gyrus	155	156	A1/2/3ulhf, area 1/2/3(upper limb, head and face region)
		157	158	A1/2/3tonIa, area 1/2/3(tongue and larynx region)
		159	160	A2, area 2
		161	162	A1/2/3tru, area1/2/3(trunk region)
Insular Lobe	INS, Insular Gyrus	163	164	G, hypergranular insula
		165	166	vIa, ventral agranular insula
		167	168	dIa, dorsal agranular insula
		169	170	vId/vIg, ventral dysgranular and granular insula
		171	172	dIg, dorsal granular insula
		173	174	dId, dorsal dysgranular insula
Limbic Lobe	CG, Cingulate Gyrus	175	176	A23d, dorsal area 23
		177	178	A24rv, rostroventral area 24
		179	180	A32p, pregenual area 32
		181	182	A23v, ventral area 23
		183	184	A24cd, caudodorsal area 24
		185	186	A23c, caudal area 23
		187	188	A32sg, subgenual area 32
Occipital Lobe	MVOcC, MedioVentral Occipital Cortex	189	190	cLinG, caudal lingual gyrus
		191	192	rCunG, rostral cuneus gyrus
		193	194	cCunG, caudal cuneus gyrus
		195	196	rLinG, rostral lingual gyrus
		197	198	vmPOS,ventromedial parietooccipital sulcus
	LOcC, lateral Occipital Cortex	199	200	mOccG, middle occipital gyrus
		201	202	V5/MT+, area V5/MT+
		203	204	OPC, occipital polar cortex
		205	206	iOccG, inferior occipital gyrus
		207	208	msOccG, medial superior occipital gyrus
		209	210	lsOccG, lateral superior occipital gyrus
Subcortical Nuclei	Amyg, Amygdala	211	212	mAmyg, medial amygdala
		213	214	lAmyg, lateral amygdala
	Hipp, Hippocampus	215	216	rHipp, rostral hippocampus
		217	218	cHipp, caudal hippocampus
	BG, Basal Ganglia	219	220	vCa, ventral caudate
		221	222	GP, globus pallidus
		223	224	NAC, nucleus accumbens
		225	226	vmPu, ventromedial putamen
		227	228	dCa, dorsal caudate
		229	230	dlPu, dorsolateral putamen
	Tha, Thalamus	231	232	mPFtha, medial pre-frontal thalamus
		233	234	mPMtha, pre-motor thalamus
		235	236	Stha, sensory thalamus
		237	238	rTtha, rostral temporal thalamus
		239	240	PPtha, posterior parietal thalamus
		241	242	Otha, occipital thalamus
		243	244	cTtha, caudal temporal thalamus
		245	246	lPFtha, lateral pre-frontal thalamus

Table T3: **Regions a-priori Affiliation**

Columns marked "R" are region numbers and "M" columns are a-priori modular affiliations. Reused from [**chinichian2023fast**]

R	M	R	M	R	M	R	M	R	M	R	M	R	M	R	M	R	M	R	M
1	1	26	11	51	6	76	2	101	15	126	13	151	9	176	4	201	5	226	3
2	1	27	7	52	11	77	1	102	15	127	13	152	9	177	9	202	5	227	3
3	7	28	11	53	12	78	8	103	13	128	14	153	4	178	4	203	5	228	3
4	11	29	14	54	12	79	2	104	13	129	14	154	4	179	4	204	5	229	3
5	4	30	14	55	12	80	6	105	5	130	14	155	12	180	1	205	5	230	3
6	4	31	14	56	12	81	7	106	5	131	8	156	12	181	13	206	5	231	3
7	1	32	11	57	12	82	15	107	14	132	8	157	2	182	13	207	9	232	3
8	1	33	6	58	12	83	6	108	14	133	14	158	2	183	1	208	9	233	3
9	1	34	11	59	12	84	15	109	15	134	14	159	14	184	1	209	14	234	3
10	1	35	6	60	12	85	6	110	15	135	13	160	14	185	4	210	14	235	8
11	1	36	6	61	2	86	6	111	13	136	13	161	12	186	4	211	15	236	8
12	1	37	1	62	2	87	6	112	13	137	7	162	12	187	4	212	15	237	4
13	4	38	1	63	14	88	6	113	13	138	11	163	8	188	4	213	15	238	4
14	4	39	6	64	14	89	15	114	13	139	14	164	15	189	5	214	15	239	8
15	1	40	1	65	13	90	15	115	15	140	14	165	1	190	5	215	4	240	3
16	11	41	4	66	8	91	14	116	15	141	8	166	1	191	10	216	4	241	4
17	7	42	4	67	12	92	14	117	15	142	11	167	1	192	10	217	4	242	3
18	11	43	6	68	12	93	15	118	15	143	6	168	1	193	5	218	4	243	3
19	1	44	11	69	15	94	15	119	13	144	6	169	8	194	10	219	3	244	3
20	1	45	15	70	15	95	7	120	15	145	8	170	8	195	10	220	3	245	3
21	1	46	15	71	2	96	15	121	6	146	8	171	15	196	5	221	3	246	3
22	11	47	4	72	2	97	14	122	6	147	13	172	15	197	10	222	3		
23	7	48	4	73	2	98	14	123	6	148	13	173	1	198	10	223	8		
24	11	49	4	74	2	99	7	124	6	149	13	174	1	199	5	224	15		
25	14	50	15	75	6	100	15	125	13	150	13	175	4	200	5	225	3		

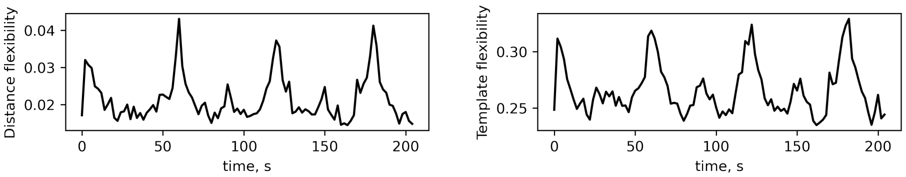

Figure S3: **Larger ensemble of shuffled DTI simulation**
An ensemble of 300 random initial conditions are used to generate these plots. Although a more regular pattern is observed in this case, both the converging rate to this regularity and range of values are smaller.

Selection of Nodes

If we sort the nodes in the DTI matrix (light to heavy i.e., low weighted degree to hight weighted degree) based on their weighted degrees, the following list of indices is achieved:

115, 117, 211, 212, 116, 119, 213, 118, 49, 238, 109, 120, 111, 114, 113, 110, 112, 187, 50, 42, 168, 188, 41, 214, 184, 174, 166, 69, 180, 195, 70, 165, 102, 47, 246, 179, 100, 96, 158, 172, 46, 183, 236, 167, 48, 242, 164, 62, 94, 92, 157, 61, 196, 171, 90, 78, 43, 231, 45, 122, 44, 40, 77, 234, 101, 232, 84, 124, 235, 215, 244, 237, 38, 37, 74, 72, 73, 189, 217, 34, 80, 163, 216, 52, 89, 98, 32, 108, 20, 153, 240, 30, 106, 51, 82, 39, 54, 105, 67, 19, 185, 76, 177, 33, 93, 147, 219, 28, 64, 103, 191, 31, 36, 228, 14, 154, 27, 245, 91, 11, **35, 173, 159, 148, 202, 146**, 79, 83, 65, 133, 192, 123, 104, 16, 178, 156, 218, 197, 13, 190, 241, 155, 88, 160, 15, 18, 95, 200, 71, 170, 149, 127, 29, 86, 24, 4, 129, 23, 151, 63, 22, 125, 220, 97, 145, 198, 66, 68, 26, 25, 204, 206, 193, 12, 134, 130, 239, 139, 194, 186, 243, 144, 21, 121, 137, 58, 128, 99, 131, 53, 201, 75, 107, 233, 87, 209, 55, 9, 132, 81, 227, 210, 181, 207, 175, 150, 1, 59, 152, 142, 85, 203, 10, 205, 135, 126, 56, 222, 223, 162, 141, 17, 8, 136, 60, 140, 199, 6, 208, 226, 161, 138, 7, 169, 224, 176, 57, 3, 2, 143, **5, 182, 221, 225, 229, 230**

Where the underlined nodes are the working memory (WM) associated nodes and the **bold** nodes at the start, middle and end, mark the three scenarios explained in the "Node Selection Scenarios" section.

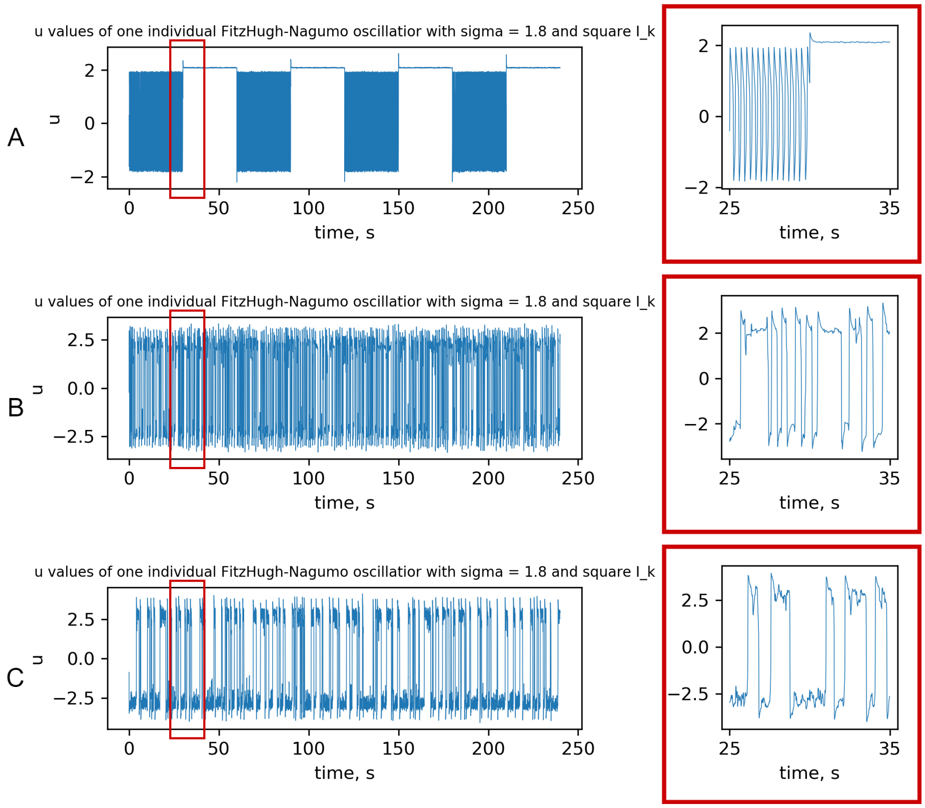

Figure S4: **Light scenario FHN timeseries**

In the left column, u time series for three different nodes (from start or low weighted degree (A), middle or intermediate weighted degree (B) and end or high weighted degree (C) of the sorted weighted degree list) are shown when 6 nodes with lightest weighted degrees (Light) are stimulated by $I_k(t)$. In the right column, a 10 seconds period with red borders is magnified.

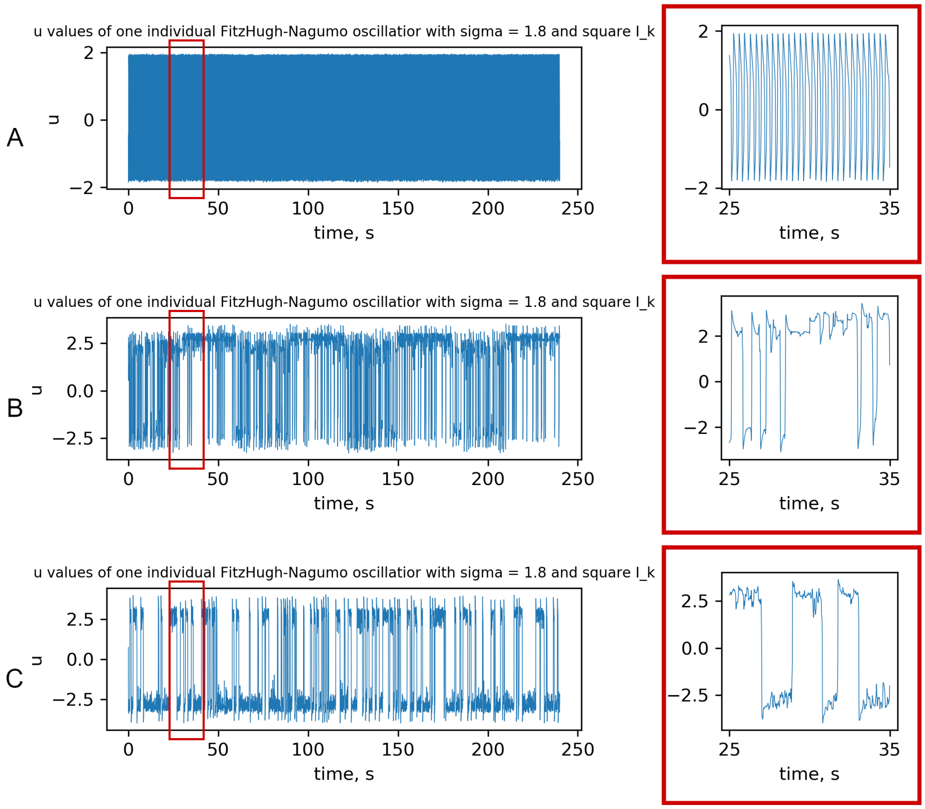

Figure S5: **Intermediate degree (Mid) scenario FHN time series** $u_i(t)$ In the left column, u time series for three different nodes (from start or low weighted degree (A), middle or intermediate weighted degree (B) and end or high weighted degree (C) of the sorted weighted degree list) are shown when 6 nodes with intermediate degree (Mid) are stimulated by $I_k(t)$. In the right column, a 10 seconds period with red borders is magnified.

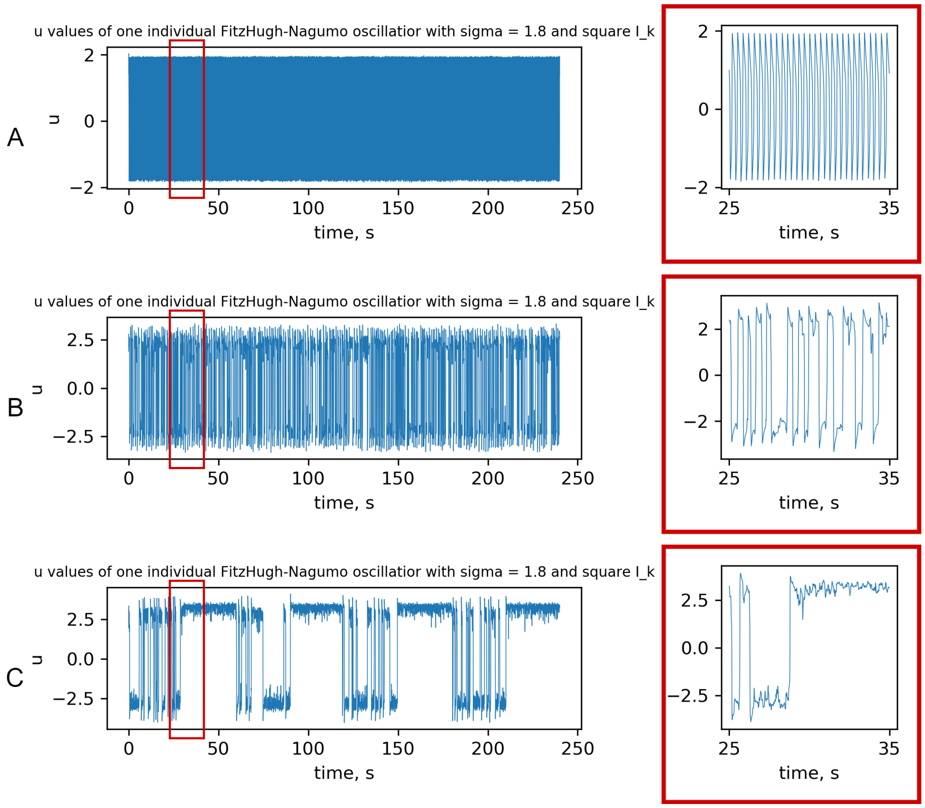

Figure S6: **Heavy scenario FHN timeseries**
In the left column, u time series for three different nodes (from start or low weighted degree (A), middle or intermediate weighted degree (B) and end or high weighted degree (C) of the sorted weighted degree list) are shown when 6 nodes with highest weighted degrees (Heavy) are stimulated by $I_k(t)$. In the right column, a 10 seconds period with red borders is magnified.

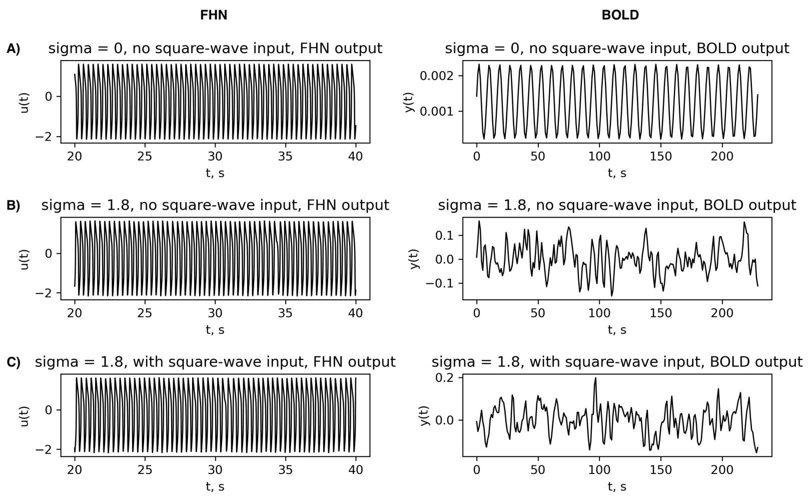

Figure S7: **FitzHugh-Nagumo and Balloon models outputs**
An example plot for the outputs of FHN and Balloon models for a region at the start of the sorted list of weighted connections (Region 115 from Brainnetome) for the 3 cases; Top: $\sigma = 0$ and no square-wave input to any region. Middle: $\sigma = 1.8$ and no square-wave input to any region. Bottom: $\sigma = 1.8$ and square-wave input of $I_k(t) = -3(2\lfloor ft \rfloor) - \lfloor 2ft \rfloor))$ given to the 6 selected working memory regions.

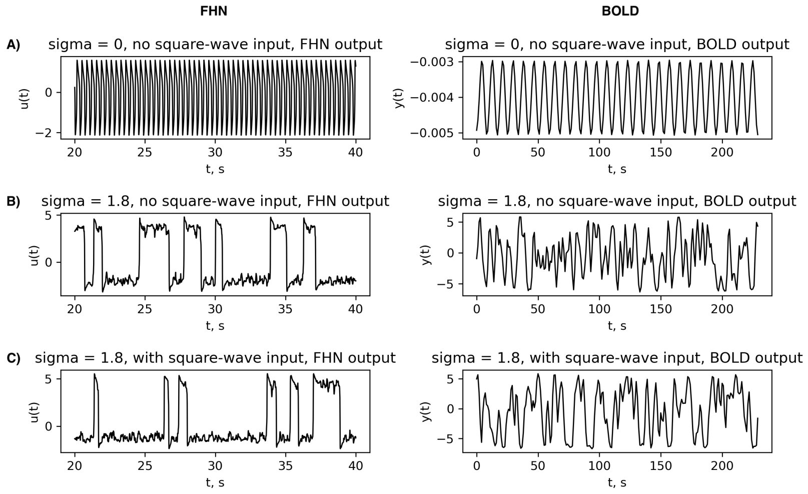

Figure S8: **FitzHugh-Nagumo and Balloon models outputs**
An example plot for the outputs of FHN and Balloon models for a region at the end of the sorted list of weighted connections (Region 230 from Brainnetome) for the 3 cases; Top: $\sigma = 0$ and no square-wave input to any region. Middle: $\sigma = 1.8$ and no square-wave input to any region. Bottom: $\sigma = 1.8$ and square-wave input of $I_k(t) = -3(2\lfloor ft \rfloor) - \lfloor 2ft \rfloor))$ given to the 6 selected working memory regions.

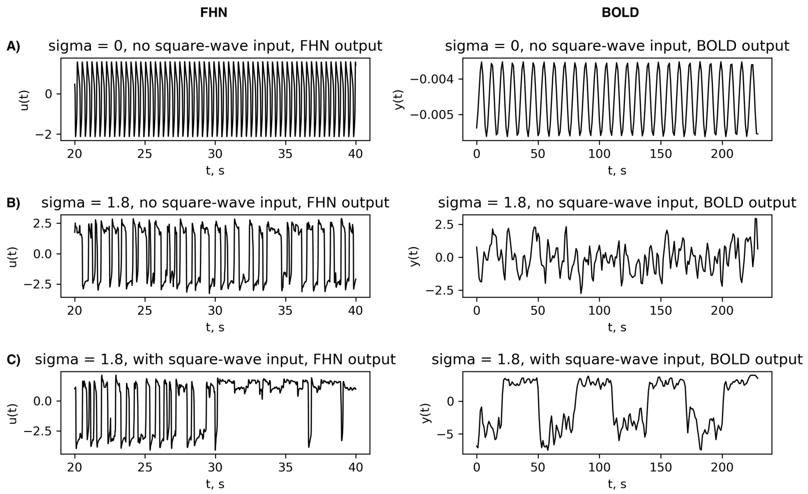

Figure S9: **FitzHugh-Nagumo and Balloon models outputs**
An example plot for the outputs of FHN and Balloon models for a region which is receiving the input I_k directly (Region 63 from Brainnetome) for the 3 cases; Top: $\sigma = 0$ and no square-wave input to any region. Middle: $\sigma = 1.8$ and no square-wave input to any region. Bottom: $\sigma = 1.8$ and square-wave input of $I_k(t) = -3(2\lfloor ft \rfloor) - \lfloor 2ft \rfloor))$ given to the 6 selected working memory regions.
